# A Method to Calibrate Chemical-Agnostic Quantitative Adverse Outcome Pathways on Multiple Chemical Data

**DOI:** 10.1021/acs.chemrestox.5c00435

**Published:** 2026-04-14

**Authors:** Zheng Zhou, Ullrika Sahlin

**Affiliations:** Department of Earth and Environmental Sciences, 5193 Lund University, Lund 22362, Sweden

## Abstract

Quantitative Adverse Outcome Pathways (qAOPs) can support next-generation risk assessment by integrating new approach methods (NAMs) for deriving points of departure. To be useful, a qAOP should be chemical-agnostic. However, existing qAOP studies often pool multichemical data without adequately addressing cross-chemical heterogeneity. Consequently, pathway relationships become obscured by heterogeneity-induced variations, thereby compromising model reliability and generalizability. We developed a calibration approach to address this challenge by leveraging hierarchical structures to systematically separate chemical-specific heterogeneity from the underlying pathway effects. Chemical-specific deviations are explicitly modeled as random effects, enabling the extraction of pathway-level parameters that represent core mechanistic relationships independent of the individual chemical properties. We demonstrated through a simulation study that performance differences between models with and without hierarchical calibration can reveal the magnitude of the heterogeneity in the data. When heterogeneity is substantial, an uncalibrated qAOP should not be considered truly chemical-agnostic in practice, as it confounds pathway-level effects with chemical-specific variations. Finally, we demonstrated the application of this calibration approach through deriving points of departure to a case study of nonmutagenic liver tumor qAOP.

## Graphical abstract

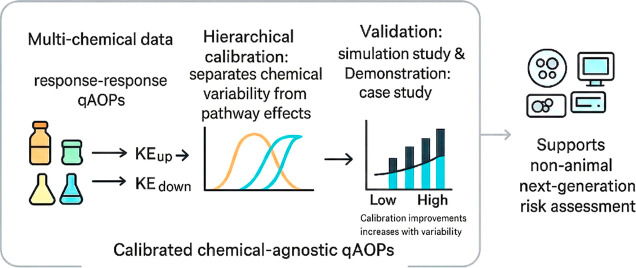

## 1 Introduction

### 1.1 Background

The Adverse Outcome Pathway (AOP) framework organizes existing knowledge on biological mechanisms that link molecular or cellular perturbations to adverse outcomes (AOs) across different levels of biological organization.^1^ It facilitates the extrapolation of mechanistic evidence from animal and nonanimal models at lower biological levels to predict AOs.^2,3^ Quantitative AOPs (qAOPs) represent an advanced stage of AOP development and application, focusing on the quantitative relationships among key events (KEs) and between KEs and AOs.^2–4^ KEs are measurable biological changes that are necessary but not sufficient for the occurrence of an AO.^1^ Robust AOPs and qAOPs are essential for implementing New Approach Methodologies (NAMs) aimed at the 3Rs—Reduction, Replacement, and Refinement—of animal testing in next-generation risk assessments.^5^ qAOPs have been increasingly applied to support hazard identification, derive points of departure (PODs), and develop integrated testing and assessment approaches for regulatory decision-making.^4,6^

### 1.2 Challenge to Achieve Chemical-Agnostic Adverse Outcome Pathways

Chemical agnosticism represents a fundamental principle of AOPs and qAOPs,^1^ effectively distinguishing them from other mechanistic frameworks such as mechanisms of action (MOAs).^7,8^ This distinction is manifested in two key aspects. First, AOPs initiate at the molecular initiating event, whereas MOAs are dose-dependent and begin with a chemical dose or exposure. Second, AOPs operate independently of specific chemicals; provided that the initial event is triggered with sufficient magnitude, the downstream chain of key events can be activated regardless of the chemical. By contrast, MOAs remain intrinsically linked to specific chemical exposures and their dose–response relationships.

However, existing qAOP studies exhibit limited adherence to the chemical-agnostic principle in two important ways. First, several studies have constructed preliminary models using data from a single chemical and a specific exposure route or measurement assay.^9,10^ Although these models may demonstrate excellent performance for the specific chemical examined, their validity for the inference of other chemicals leading to the same AO remains unverified. This lack of generalizability significantly restricts the applicability of such qAOP models for the prospective prediction of new chemicals in regulatory and industrial contexts. Second, some qAOP studies have developed models using multichemical data sets while inadequately addressing cross-chemical heterogeneity.^11–13^ Such heterogeneity arises from various factors, including differences in chemical structures and properties,^1^ which can lead to substantial variation in the potency required to trigger upstream key events across chemicals. When heterogeneity is pronounced, a dose sufficient to induce the complete cascade of key events and the adverse outcome in one chemical may fail to trigger the initial event in another. Therefore, neglecting this heterogeneity undermines the fundamental chemical-agnostic principle and compromises model reliability across diverse chemical exposures. When data exhibiting substantial cross-chemical heterogeneity are pooled without accounting for such differences, the pooled model will differ significantly from the models derived on individual chemicals and may be biased by extreme data points.^14^

### 1.3 Study Objectives

Given the importance of considering heterogeneity in multichemical data sets, it is essential to distinguish chemical-specific heterogeneity from the underlying chemical-agnostic pathway when developing and applying qAOPs. Hierarchical modeling is an established statistical parametric approach for considering grouped data,^14,15^ which has been successfully applied to dose–response modeling.^16,17^ However, the implementation of hierarchical structures in qAOP models, particularly parametric response–response models, remains largely unexplored. By incorporating hierarchical frameworks into qAOP development, it is possible to explicitly account for cross-chemical variability while preserving the chemical-agnostic principles fundamental to the AOP framework. This study addresses this methodological gap by developing and evaluating a hierarchical response–response model for multichemical qAOP applications.

The aim of this study is to develop a framework to calibrate chemically agnostic qAOP models to facilitate more robust and generalizable qAOPs. Specifically, the objectives are to (1) promote the incorporation of multichemical data in qAOP development, (2) develop a modeling approach that can effectively separate chemical-specific heterogeneity from chemical-agnostic pathways using hierarchical structures, and (3) evaluate the predictive performance of the qAOP with hierarchical structures against the one without.

## 2 Theory and Methods

### 2.1 Quantitative Adverse Outcome Pathway Model

The following qAOP model is considered for the chemical-agnostic calibration of this study: the upstream key event, denoted as KE_up_, is modeled as a continuous random variable *Y*_1_; the downstream key event, denoted as KE_down_, is modeled as a dichotomous random variable *Y*_2_. The Benchmark Dose (BMD) methodology is employed to model the dose–response relationship between dose and *Y*_1_ and the response–response relationship between *Y*_1_ and *Y*_2_. We modified the model from BMDS guidance^18^ and EFSA guidance^19^ to eq 1: (1) the outcome of KE_up_ follows a log–normal distribution, where at any given doses, the expected value of *Y*_1_ for individual *i* = 1, ...*n*_*j*_ in group *j* on the log scale is dependent on a latent group mean, with a standard deviation σ_1_ representing the variation across individuals in the group. (2) Based on the results of a constant coefficient of variation test, σ_1_ could be constant or dose-dependent.^18,19^ (3) The latent group mean *M* is predicted by a functional form which depends on dose *d* and parameters θ_1_, with a standard deviation σ_2_ representing the residuals not captured by the functional form. The outcome of KE_down_ follows a Bernoulli distribution, where for any given *Y*_1_, the probability of *Y*_2_ is dependent on *Y*_1_ and parameters θ_2_.

1 $$\begin{array}{ll} Y_{1_{ij}}|M_{j},\sigma_{1} & ∼\ln\left(M_{j},\sigma_{1}\right)\\ M_{j}|d_{ij},\theta_{1},\sigma_{2} & ∼N\left(f_{1}\left(d_{ij},\theta_{1}\right),\sigma_{2}\right)\\ Y_{2_{ij}}|Y_{1_{ij}},\theta_{2} & ∼Be\left(f_{2}\left(e^{M_{j}},\theta_{2}\right)\right) \end{array}$$

When the data is presented in a summarized format, for a dose group *j* = 1, ..., *J*, the dose–response data includes the group mean, standard deviation, and size as *m*_*j*_,*s*_*j*_,*n*_1*j*_, respectively, and the response–response data includes the group count of cases and total number of subjects as *X*_*j*_,*n*_2*j*_, respectively. These summarized data are treated as sufficient statistics so that the model becomes eq 2: (1) the lognormality assumption for KE_up_ holds, while *Y*_1*j*_ becomes *m*_*j*_,*s*_*j*_,*n*_1*j*_. The constant coefficient test also applies to σ_1_. (2) Summarized KE_down_ follows a binomial distribution, and the count of cases *X*_*j*_ is the sum of independent Bernoulli trials among *n*_2*j*_ subjects and depends on the latent population mean *M*_*j*_ and parameters θ_2_.

2 $$\begin{array}{ll} m_{j},s_{j},n_{1_{j}}|M_{j},\sigma_{1} & ∼\ln\left(M_{j},\sigma_{1}\right)\\ M_{j}|d_{j},\theta_{1},\sigma_{2} & ∼N\left(f_{1}\left(d_{j},\theta_{1}\right),\sigma_{2}\right)\\ X_{j}|n_{2_{j}},Y_{1_{j}},\theta_{2} & ∼Binom\left(n_{2_{j}},f_{2}\left(e^{M_{j}},\theta_{2}\right)\right) \end{array}$$

The log-likelihood function for KE_up_ is provided on the summarized data *n*_1_, *m*, *s*, and *d* at log scale and parameters θ_1_ and σ_1_:

3 $$\begin{array}{l} LL\left(N,n_{1_{j}},m_{j},s_{j},d_{j};M_{j},\sigma_{1}\right)\\ =-\frac{N}{2}\log\left(2\pi \right)-\sum_{j=1}^{G}\frac{n_{1_{j}}}{2}\log\left(\sigma_{1}^{2}\right)+\frac{n_{1_{j}}{\left[m_{j}-M_{j}\right]}^{2}+\left(n_{1_{j}}-1\right){s_{j}}^{2}}{2\sigma_{1}^{2}} \end{array}$$

where *N* is the total number of observations. Since lognormality is assumed, the summarized group mean *m*_*Y*_ and standard deviation *s*_*Y*_ at regular scale can be transformed to approximate *m* and *s* using Equation S5 in Section 5 of SI.

The log-likelihood for KE_down_ is provided on the summarized data *n*_2_, *X*_2_, and *M* and parameters θ_2_:

4 $$LL\left(n_{2_{j}},X_{j},M_{j},\theta_{2}\right)=X_{j}\log\left(f_{2}\left(e^{M_{j}},\theta_{2}\right)\right)+\left(n_{2_{j}}-X_{j}\right)\log\left(1-f_{2}\left(e^{M_{j}},\theta_{2}\right)\right)$$

The qAOP evaluates the sum of the two log-likelihoods. For details on the application of benchmark dose modeling, we recommend the User Guide^20^ and Technical Guidance^18^ of the U.S. EPA Benchmark Dose Software (BMDS) and EFSA guidance.^19^

We used a continuous-Hill function from BMDS^18^ (Equation 5) with parameters θ_1_ = {*a*, *b*, *c*, *g*} as the functional form of *f*_1_, where *a* is background response; *b* is maximum change, *b* > 0 for increasing and *b* < 0 for decreasing trends, respectively; *c* > 0 is potency, i.e., the dose at which half-maximal change occurs; and *g* > 0 is power, i.e., the steepness of sigmoid curves.

5 $$f_{1}\left(d,\theta_{1}\right)=a+b\frac{d^{g}}{c^{g}+d^{g}}$$

For the response–response part, the dichotomous Hill function from the BMD guidance^19^ was reparametrized for *f*_2_ (Equation 6) with parameters θ_2_ = {*v*, *q*, *h*, *r*}, where 0 < *v* ≤ 1 is maximum probability; 0 ≤ *q* < 1 is the background risk ratio so that *vq* is background probability; *h* > 0 is potency, i.e., the level resulting in half-maximal effect; and *r* is power, i.e., slope of sigmoid curves, *r* > 0 for increasing trends and *r* < 0 for decreasing trends.

6 $$f_{2}\left(e^{M_{j}},\theta_{2}\right)=v\frac{1+qe^{rM_{j}-h}}{1+e^{rM_{j}-h}}$$

Since the focus here is on evaluating the calibration approach rather than finding the best model, the Hill functions are used because they are flexible and biologically relevant.^18,19,21^ The flexibility of Hill functions allows the modeling of a potential threshold in the dose–response and response–response relationship.

### 2.2 Hierarchical Structure

For each chemical *k* = 1, ..., *K*, there are *G*_*k*_ dose groups, denoted as *j* = 1···*G*_*k*_. A “*flat*” response–response model, i.e., without hierarchical structure, is the dichotomous Hill function in eq 6 with the same set of parameters θ_1_ = {*v*, *q*, *h*, *r*} for all chemicals. In contrast, a “hierarchical” model allows parameter values to vary across chemicals. Identical parametrization is employed for the dose–response component of both flat and hierarchical models to control for uncertainty arising from dose–response modeling. For better flexibility, dose–response parameters *a*, *b*, *c*, and σ_1_ are made chemical-specific for both the flat and hierarchical models, while parameters *g* and σ_2_ are left as global. These chemical-specific dose–response parameters do not affect the definition of a flat model because the distinction between flat and hierarchical models in this study is strictly based on the difference in the response–response part. By maintaining consistency in the dose–response modeling approach, we can attribute any differences in predictive performance between the flat and hierarchical models solely to structural differences in the response–response calibration. This design allows for a direct comparison of model performance and isolates the impact of incorporating hierarchical structures. Full model scripts are available in SI. The hierarchical structure incorporates two key features (Equation 7).

7 $$f_{2}\left(e^{M_{j}},\theta_{2}\right)=v_{k}\frac{1+q_{k}e^{rM_{j}-h_{k}}}{1+e^{rM_{j}-h_{k}}}$$

First, parameters are assigned with hierarchical levels based on their mechanistic interpretation: parameters *v*, *q*, and *h* are chemical-specific, while *r* is chemical-agnostic. Parameters *v* and *q* are the maximum probability and maximum ratio, which represent the upper bound for response and the ratio of upper to lower bounds for response, respectively. Consequently, making them chemical-specific is justified on the grounds that it is not uncommon for the upper and lower bounds to vary across chemicals. Furthermore, the upper and lower bounds could be correlated per chemical, which supports the multivariate joint distribution on them. Parameter *h* is the potency, which stands for the level of KE_up_ inducing half of the observed maximum KE_down_. It is defined in this study to be chemical-specific to reflect a fundamental assumption in the AOP chemical-agnostic principle^1^ that chemicals described by the same AOP follow a similar path of KE progression, while the level of progression may vary across chemicals. Moreover, chemical-specific potency has been suggested and employed in previous studies to allow the derivation of a relative potency factor and comparison across chemicals.^22^ Parameter *r* is defined as chemical-agnostic, which is justified on two grounds. From a mechanistic perspective, it controls the shape and steepness of the sigmoid region of the response–response curve. The overall shape is governed by the pathway and supposed to be shared among chemicals. Moreover, empirical evidence from previous meta-dose–response studies demonstrates that estimated log-inflection points and steepness parameters exhibit minimal variation across chemicals, even when modeled individually.^22,23^

Second, heterogeneity is parametrized through a combination of random and fixed effects. Specifically, the structure of each hierarchical parameter comprises three components (Equation 8): (a) a chemical-agnostic central location, (b) chemical-agnostic variability, and (c) chemical-specific relative deviations from the central location. The product of the chemical-agnostic variability and chemical-specific relative deviations constitutes the random effects that capture departures from the central pathway for each chemical.

8 $$\begin{array}{ll} v_{k} & =\mu^{v}+\sigma^{v}z_{k}^{v}\\ q_{k} & =\mu^{q}+\sigma^{q}z_{k}^{q}\\ \kappa^{vq} & =\left(\mu^{v},\mu^{q}\right)\\ \Sigma^{vq} & =var\left(\sigma^{v},\sigma^{q}\right)\\ L^{vq} & =corr\left(z_{j}^{v},z_{j}^{q}\right)\\ \left(v,q\right) & ∼MVN\left(\kappa^{vq},\Sigma^{vq}L^{vq}\right)\\ h & =\mu^{h}+\sigma^{h}z_{k}^{h} \end{array}$$

where a superscript indicates the dependent, low-level parameter; μ and σ are chemical-agnostic central location and variability, respectively; *z*_*k*_ are chemical-specific relative deviations; and MVN refers to multivariate normal distributions, which depend on a mean vector κ^*vq*^, a standard deviation matrix Σ^*vq*^, and a correlation matrix *L* ^*vq*^.

### 2.3 Model Performance

The impact of the hierarchical calibration approach is evaluated by comparing the predictive performance of the flat-line to the hierarchical model specification. Model performance is assessed using the expected log predictive density (ELPD, Equation 9), which enables comparison of posterior predictive performance between non-nested, nonlinear models,^24,25^ and the Widely Applicable Information Criterion (WAIC)^25^ to check the robustness toward the choice of metric. The ELPD is defined as

9 $$elpd\left(y,{ỹ}_{i}\right)=\sum_{i=1}^{n}\int p_{t}\left({ỹ}_{i}\right)\log p\left({ỹ}_{i}|y\right)d{ỹ}_{i}$$

where $p_{t}\left({ỹ}_{i}\right)$ is the distribution for the true data-generating process for ${ỹ}_{i}$ . In this study, $p_{t}\left({ỹ}_{i}\right)$ is approximated by Pareto Smoothed Importance Sampling Leave-one-out Cross Validation (PSIS–LOO–CV),^26^ implemented using the loo package in R.^27^ For brevity, elpd_PSIS–LOO_ is hereafter referred to as LOO. According to the definition of LOO,^25,26^ if LOO_hierarchical_ exceeds LOO_flat_, the hierarchical model demonstrates superior predictive performance compared to the flat model. Such a result indicates that cross-chemical heterogeneity in the data is sufficiently large to warrant the use of hierarchical structures without causing overfitting. Conversely, if the two models exhibit comparable LOO values, the added complexity of the hierarchical structure may not be justified for the data set under consideration.

### 2.4 Data Requirements

To characterize the effect of dose on both upstream and downstream key event responses, the calibration framework requires (1) two dose–response data sets corresponding to the upstream and downstream key events, respectively, and (2) some chemicals shared between these two data sets. High-quality data are essential for robust model development and validation. The highest quality toxicological data originate from randomized controlled trials, which provide unbiased estimates of dose–response relationships.^6^ When experimental data are unavailable, data from observational studies may be considered as an alternative. However, observational data carry substantial risks of bias, resulting in lower weight of evidence^6,7^ and potentially compromising the robustness of the qAOP framework. To enable meaningful characterization of cross-chemical heterogeneity, both data sets must include multiple shared chemicals.

Data quality can be further enhanced when both data sets originate from the same experiment, such that they naturally share the same applicability domain (such as an *in vivo* route of exposure or an *in vitro* assay) and dosage specifications (i.e., dose groups), thereby ensuring highest comparability. Conversely, when the two data sets are collected from separate experiments or studies, the shared chemicals must have identical or at least comparable applicability domains to maintain validity. In such cases, complete alignment of dosage setups across the original data sets is not strictly necessary as long as doses are converted to consistent units compatible with the qAOP framework. A latent variable *Y*_1_|*d*_2_,θ_1_ is required and computed on the estimated values of θ_1_ and *d*_2_ from the KE_down_ data to bridge the two log-likelihoods.

Since it is not uncommon in existing toxicological studies to have summarized data, the framework in this study is tailored for the summarized format for better generalizability. However, from the perspective of robustness, access to individual-level data brings unique advantages since aggregation results in information loss. With individual-level data, it becomes feasible to evaluate the magnitude of within-group variability and determine whether it is necessary to separate it from between-group variation and whether a homogeneous variation parameter remains appropriate. Preserving the highest resolution of data whenever possible enhances the reliability and precision of the hierarchical calibration approach.

### 2.5 Model Implementation

Model calibration was done through Bayesian updating using a No–U-Turn Sampler in Hamiltonian Monte Carlo sampling through the *R* interface of Stan.^28^ The target log-density functions and complete Stan scripts are available in SI Section 8.

Weakly informative priors were assigned to all model parameters to limit the prior influence while maintaining computational stability (Table 1). In particular, we avoid using uniform distributions for priors, based on the fact that for some chosen ranges on benchmark dose parameter, this can result in bias in the derived point of departure.^29^

**Table 1:** Prior Distributions for the Parameters of the Chosen Models in the Statistical Calibration

dose–response function		response–response function, flat		response–response function, hierarchical	
parameter name	distribution	parameter name	distribution	parameter name	distribution
a	normal	*v*	beta	κ_*vq*	normal
b	normal	*q*	beta	Σ_*vq*	cauchy
c	normal	*z*_vq	multivariate normal	L_vq	lkj_cholesky
g	normal	*h*	normal	z_vq	normal
		*r*	normal	μ_*h*	normal
				σ_*h*	cauchy
				*r*	normal

Here, probability distributions and hyperparameters for priors were specified using a simple iterative prior predictive evaluation procedure to ensure they yielded plausible predictions within the context of the AO.^30^ For the case study, a set of hyperparameter values was initially drawn from the specifications of the source study, and the corresponding prior predictive distribution was generated. The resulting distribution was evaluated by comparing its range and shape against the empirical observations of tumor incidences, which were bounded between 0 and 1. Hyperparameters were iteratively adjusted until the prior predictive distribution adequately spanned the full data range and exhibited realistic variability without excessive concentration in any subregion of the response space. The final set of hyperparameter values used in the analyses is provided in SI Section 4.

The MCMC sampling procedure comprises four chains, each consisting of 10,000 warm-up iterations and 50,000 sampling iterations. The performance of the MCMC samplers is evaluated for chain convergence and sampling efficiency using the R̂ statistic and effective sample size (ESS), respectively. Convergence is deemed satisfactory when R̂s are close to 1 for all parameters, indicating that the chains have adequately explored the posterior distribution. Additionally, adequate sampling efficiency is confirmed when the ESS exceeds 400 for all parameters, ensuring sufficient independent samples for reliable posterior inference.

### 2.6 Simulation Study

A simulation study was conducted to validate the calibration framework across varying levels of cross-chemical heterogeneity. The simulation procedure is illustrated in Figure 1. To isolate the impact of hierarchical calibration on the response–response component, both flat and hierarchical models employ identical dose–response models, thereby ensuring that any performance differences can be attributed solely to the calibration on the response–response part. Complete scripts for the simulation study and subsequent analyses are provided in SI Section 9.

**Figure 1:**
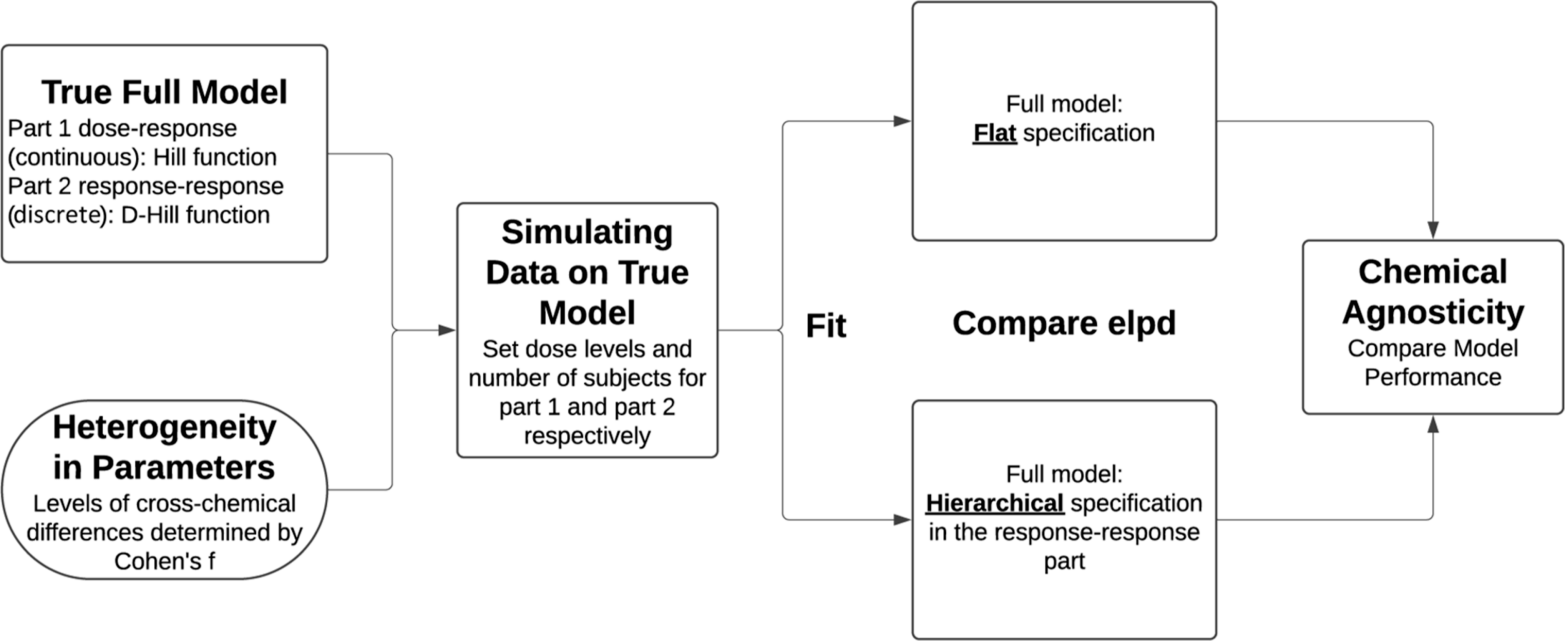
Overview of the simulation study.

Data sets for the simulation study were generated at four levels of cross-chemical heterogeneity (none, small, medium, and large) by increasing the variance of the random terms in the hierarchical model. To verify heterogeneity levels, the ratio of cross- and within-chemical variance, adjusted for doses, was calculated as Cohen’s f effect size^31^ following eq 10. A large effect size indicates that the changes in KE_down_ across chemicals are bigger than that of the same chemical over KE_up_.

10 $$\begin{array}{l} ES=\sqrt{\frac{SSB}{SST-SSB}}\\ SSB=\sum_{j=1}^{K}n_{j}{\left(\mu_{j}-\mathrm{\mu ̂}\right)}^{2}\\ SST=\sum_{i=1}^{N}{\left(y_{i}-\mathrm{\mu ̂}\right)}^{2} \end{array}$$

where SSB and SST are between-chemical and total squared variance, respectively; μ̂ is the global mean of the data; *j* is the chemical index; and *n*_*j*_ and μ_*j*_ are the size and mean per chemical, respectively. Response–response parameters were iteratively adjusted until the computed Cohen’s f values of the simulated data sets matched the true heterogeneity levels, following established thresholds:^31,32^ none = 0.001, small = 0.1, medium = 0.2, and large ≥0.4. Detailed descriptions and contextual justification for these threshold values are provided in SI Section 3. By doing so, each simulation scenario represents a distinct and interpretable level of cross-chemical heterogeneity, enabling systematic evaluation of the hierarchical calibration framework’s performance across varying data structures.

The simulated data consisted of doses, responses of KE_up_, and responses of KE_down_ for each chemical. These data sets were fitted separately using both flat and hierarchical model specifications. The difference in predictive performance between the two specifications was evaluated by using the differences in their LOO and WAIC values. To ensure the stability of results across different response–response parameter values, the simulation procedure was repeated 100 times at each heterogeneity level with performance metrics averaged across all iterations.

### 2.7 Case Study: Nonmutagenic Liver Tumors Induced by Hepatic Proliferation

A case study was included to demonstrate the application of the calibration approach. The case study utilizes multichemical rodent data from a previous study^33^ on nonmutagenic tumorigenesis induced by sustained hepatic cell proliferation. These data are supported by a key event relationship “*Induction, Sustained Cell Proliferation leads to Formation, Liver tumor*” (Key Event Relationship ID 298 ^34^) in the AOP of “*Inhibition of iNOS, hepatotoxicity, and regenerative proliferation leading to liver tumors*” (AOP ID 32 ^35^). Multiple doses per chemical were administered to male and female Fisher F344 and Sprague–Dawley rats. Sustained liver cell proliferation was quantified as the percentage of BrdU-positive cells using the 5-bromo-2-deoxyuridine (BrdU) incorporation assay.^36^ Extrahepatic tumor incidence in rats was computed from tumor incidence after adjusting for background incidence. Detailed experimental protocols and quality control procedures are available in the original publication.^33^ The complete data set comprises two dose–response data sets in summarized format for BrdU(%) (KE_up_) and tumor incidence (KE_down_) of 10 chemicals from 11 experimental studies and is provided in SI Section 5 (methapyrilene hydrochloride was tested by two studies). This data set satisfies the essential requirements raised in Section 2.4, including internal liver doses (ng/g), BrdU measurements, and tumor incidence values for each dose group and chemical. An exploratory data analysis was performed on the case study data to examine the dose–response and response–response patterns.

The data were fitted using two models based on the qAOP framework (Equation 6): one with a flat structure and one with a hierarchical structure. To isolate the impact of hierarchical calibration, both models employ identical dose–response structures, thereby controlling for uncertainty arising from dose–response modeling. Predictive performance was evaluated based on the models’ ability to predict both dose–response and response–response relationships, providing a comprehensive assessment of model accuracy across the entire pathway.

## 3 Results and Discussion

### 3.1 Results of the Simulation Study

The response–response data generated through the iterative parameter adjustment procedure are visualized in Figure 2, illustrating how the true heterogeneity level determines parameter values and controls the shapes and locations of the simulated curves. In the absence of heterogeneity, parameters were concentrated around single point values to achieve Cohen’s f = 0.05, resulting in nearly identical response–response curves across all chemicals. At the small heterogeneity level, parameter values exhibited modest dispersion, yielding simulated data with Cohen’s f = 0.1. This level produced homogeneous curve shapes across chemicals with only slight variations in the location. When heterogeneity increased to the medium level (Cohen’s f = 0.2), both the shapes and locations of the curves began to diverge noticeably across chemicals. At the large heterogeneity level, the simulated data achieved Cohen’s f = 0.5, with response–response curves exhibiting substantial variation in shape, location, and range across chemicals. These systematic differences demonstrate that the simulation procedure successfully generates data sets spanning a wide spectrum of cross-chemical heterogeneity, enabling robust evaluation of the hierarchical calibration framework.

**Figure 2:**
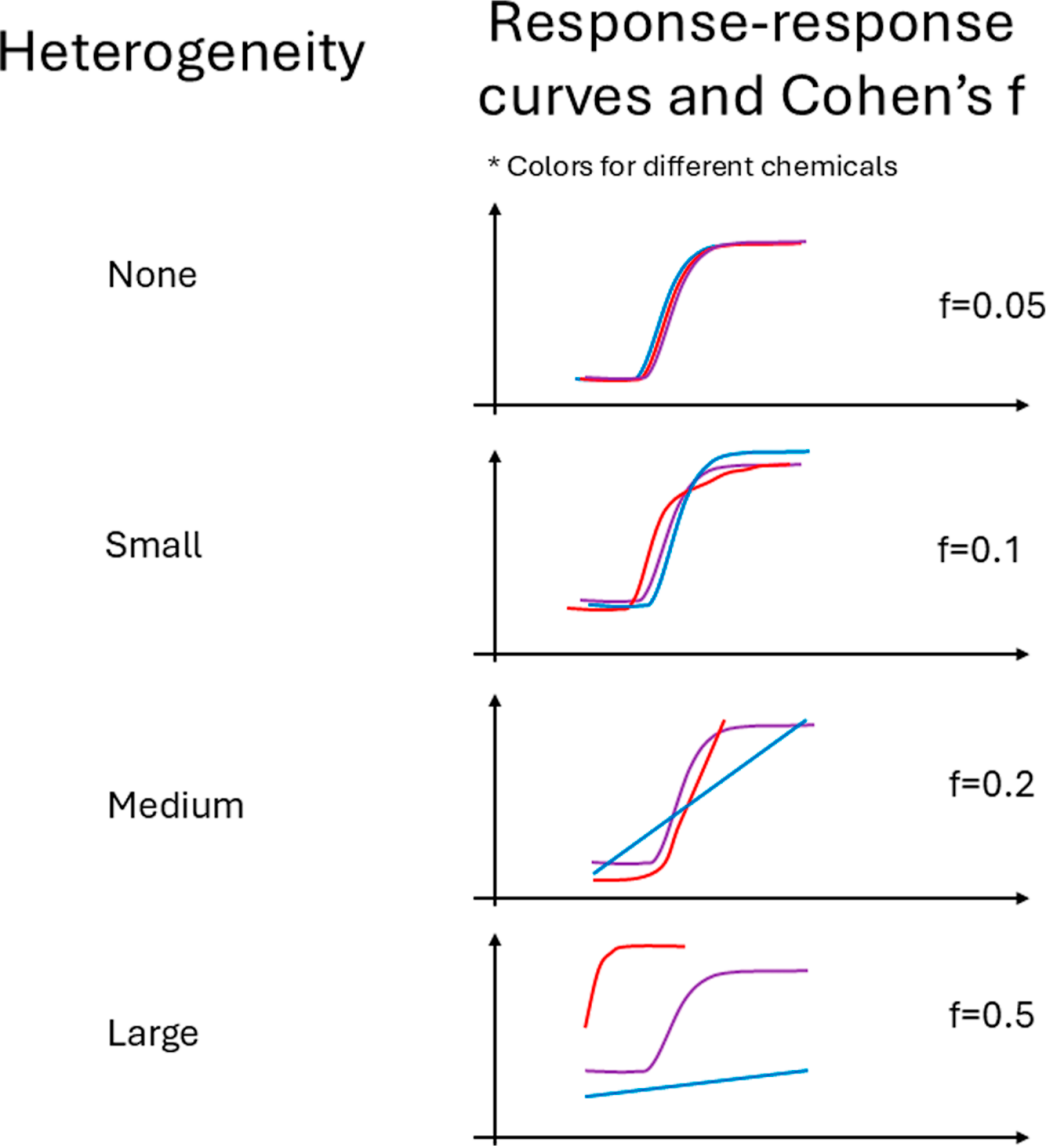
Diagram comparing response–response data of varying Cohen’s f.

After fitting both models to the simulated data sets, the model performance metrics (WAIC and LOO) demonstrated that the advantage of the hierarchical model over the flat model increases with the level of heterogeneity (Table 2). The comparative analysis revealed several key patterns. First, when heterogeneity exceeds the small level, both metrics consistently favor the hierarchical model. Under these conditions, the flat model conflates chemical-agnostic pathway effects with heterogeneity-induced variation, compromising the predictive performances. Conversely, when heterogeneity is minimal (none or small), the flat model may perform comparably or even slightly better due to better parsimony. However, as heterogeneity increases, the flat model’s inability to separate cross-chemical variation from underlying pathway effects leads to substantially degraded performance. Second, WAIC and LOO exhibited modestly divergent preferences for the hierarchical model at the medium heterogeneity level. Although both metrics are appropriate for Bayesian model comparison, LOO is generally preferred over WAIC^25^ because they handle predictive performance evaluation and penalization of overparametrization differently. In this study, the use of noninformative priors rendered the posterior distributions largely data-driven, with a limited number of extreme observations. As WAIC relies on variance-based approximations computed from the full data set, it is more susceptible to the influence of such extremes. In contrast, the LOO evaluates predictive performance by assessing changes in the posterior when individual observations are omitted, yielding a more robust basis for model comparison under these conditions. Overall, the performance differential between the flat and hierarchical models accurately reflects the underlying level of cross-chemical heterogeneity in the simulated data. These results provide strong empirical support for the validity of the proposed hierarchical calibration methodology.

**Table 2:** Comparison of Predictive Performances between Flat and Hierarchical Models across Different Heterogeneity Levels

		WAIC		LOO		
hetero	Cohen’s f	Diff^a^	Prop.Hier^b^	Diff^a^	SE	Prop.Hier^b^
0 = None	0.05	–0.52(1.83)	3%	–5.70(2.66)	1.85(0.69)	8%
1 = Small	0.1	–0.06(3.68)	42%	–0.99(1.96)	1.75(0.36)	44%
2 = Medium	0.2	1.22(1.82)	66%	5.51(3.09)	2.15(0.76)	94%
3 = Large	0.5	9.34(2.73)	100%	12.3(4.01)	4.04(1.29)	100%

a Mean and standard deviation (in parentheses) over iterations; positive values indicate support for the hierarchical model.

b Proportion of iterations supporting the hierarchical model.

### 3.2 Results of the Case Study

For the case study data, the true heterogeneity level is unknown. An exploratory data analysis shows that 71% and 24% of response–response data are predominantly concentrated in the low (log-BrdU ≤0.5) and medium (0.5–2) KE_up_ ranges, with 5% of the data available in the high KE_up_ range (log-BrdU ≥2) (SI Figure S3). The spatial distribution of the response–response data resembles the graphical pattern of medium heterogeneity in Figure 2. The majority of data in the low KE_up_ range exhibit minimal variation across chemicals, although two data points from chemicals 7 and 9 fall outside the primary concentration area. In the high KE_up_ range, two points from chemicals 3 and 11 display log-BrdU values (∼0.25) substantially lower than other points in this range (∼0.8–0.9). The upper bounds of the probability of *Y*_2_ vary significantly across chemicals. The difference between the two studies (3 and 4) on methapyrilene hydrochloride is significant to justify the use of the data as two separate chemical clusters. These observations are consistent with the empirical Cohen’s f value of 0.17, indicating a small to medium ratio of between- to within-chemical variance. Furthermore, results of a constant variation test^18^ show that a constant coefficient of variation holds for BrdU because the per group standard deviation is proportional to the per group mean (visualized in Figure S4). This evidence supports the hierarchical parametrization of chemical-specific *v*, *q*, and *h*, which is expected to demonstrate superior performance compared to the flat model.

This expectation was confirmed by the model comparison results, which modestly favor the hierarchical model (WAIC_diff = 10.1(4.0) and LOO_diff = 9.1(3.6)). This indicates that cross-chemical differences in the case study data are sufficiently large and that the flat model failed to adequately capture this heterogeneity. Consequently, the flat model cannot be considered truly chemical-agnostic. The estimated parameter values for both the flat and hierarchical models are provided in the Supporting Information file.

A detailed graphical examination of the fitting results revealed additional insights. First, although the shapes are similar, the ranges of chemical-specific random effects are large (Figure S5). This provides a visualization for the magnitude of the low-to-medium cross-chemical heterogeneity. Second, both models fit the data well in the low KE_up_ range, where the data concentration is the highest. The predicted curves from the two models are nearly indistinguishable in this range, with the hierarchical model curve (blue dotted line) positioned slightly left of the flat model curve (red solid line). However, last but not least, differences emerge in the high KE_up_ range. Although the mean curves remain similar, the 95% probability intervals of the hierarchical model (blue ribbon) are wider and provide substantially better coverage of the data compared with the flat model (red ribbon). Specifically, the hierarchical model exhibits broader upper and lower bounds that accommodate data points from chemicals 1, 3, 4, 5, and 7. This is because when propagated through the nonlinear functions, big differences in values may get scaled down to a small variance. The flat model underestimates parameter variability by pooling all data in the Hill function. In contrast, the hierarchical model correctly characterizes the heterogeneity as random effects in the Hill function and achieves better estimation of parameter variability.

Since the dose–response components of both models were identical and prior distributions were the same, all observed performance differences can be attributed to the response–response model structure. Therefore, the superior predictive performance of the hierarchical model results directly from the hierarchical structures implemented through the proposed calibration approach. Although the purpose of the case study here is not to find the best response–response model, it is crucial to recognize the importance of functional forms. The impact of the choice of functional form and parametrization on the calibration was analyzed by extending the above comparison of flat and hierarchical models on an alternative functional form for the response–response part (details in SI Section 7). Despite the changes in the functional forms and parametrizations, the hierarchical model displayed consistent advantage over the flat model. Therefore, it suggests that at a given data heterogeneity level, the impact of calibration with hierarchical models could be independent of the choice of functional forms and parametrizations.

Overall, these results demonstrate that the heterogeneity in the case study data is sufficiently substantial that the flat model violates the chemical-agnostic principle, whereas the hierarchical framework provides a valid methodological approach for identifying and characterizing cross-chemical heterogeneity.

### 3.3 Demonstrating Application with Case Study Results

To demonstrate the pragmatic application of the chemical-agnostic calibrated qAOP, we computed benchmark dose values for KE_up_ with a benchmark response (BMR) of an extra 5% increase in tumor incidence, following eq 11 from the U.S. EPA^18^ and EFSA guidance.^19^

11 $$BMR=\frac{f\left(BMD\right)-f\left(0\right)}{1-f\left(0\right)}$$

BMD values were calculated from the estimated model parameters as the doses corresponding to a predetermined benchmark response (BMR) level. For the hierarchical model, a BMD distribution was computed by using only the chemical-agnostic parameters. The derived values are hereafter referred to as benchmark “levels” (BMLs) rather than “doses” to emphasize that they are derived from BrdU rather than internal liver doses in a response–response qAOP framework.

The mean, lower bound, and upper bound of the computed BMLs are presented in Table 3 as BML, BMLL, and BMLU, respectively. The table also includes the ratio of mean to lower bound values (BML/BMLL), which provides two important insights.

**Table 3:** Benchmark Levels of BrdU % (Regular and Log Scale) on Tumor Incidence Comparing Flat and Hierarchical Model Estimates

model		flat	hierarchical
BrdU	BML	1.24	1.08
	BMLL	1.12	1.09
	BMLU	1.41	1.14
Ln(BrdU)^a^	BML	0.21	0.09
	BMLL	0.11	0.07
	BMLU	0.34	0.13
	BML/BMLL	1.91	1.12

a Same scale as the *x*-axis in Figure 3.

First, the hierarchical model yields a lower BML/BMLL ratio (1.12) compared with the flat model (1.91), corroborating the previously reported performance metrics (WAIC_diff and LOO_diff). The BML/BMLL ratio reflects the level of uncertainty between mean and lower bound estimates attributable to both data heterogeneity and model performance. A model is considered empirically reliable if this ratio falls within the range of 1–3,^20^ and for model comparison purposes, lower ratios indicate superior performance.^20^ Since both models are fitted to the same data set, uncertainty arising from data heterogeneity is held constant. Consequently, the hierarchical model’s lower BML/BMLL ratio demonstrates superior characterization of uncertainty compared to the flat model.

Second, the BML, BMLL, and BMLU values from the hierarchical model are consistently lower than those from the flat model at a 5% BMR. These differences remain below 2-fold, consistent with the relatively small to medium heterogeneity among chemicals in the low KE_up_ range. This finding aligns with the graphical patterns shown in Figure 3, where the hierarchical model curve is positioned to the left of the flat model curve. From a regulatory perspective, the conservative choice for the point of departure in risk assessment^19,20^ would be the lower BML (or BMLL) value derived from the hierarchical model. It is important to note that these differences in the BML values should not be interpreted as direct indicators of model performance. According to EPA BMDS guidelines, a model yielding lower BMD values should not be recommended if its performance metrics (e.g., AIC in BMDS) are inferior to those of alternative candidate models.^18^ In this case study, however, the hierarchical model demonstrates both superior performance metrics and lower, more conservative BML values. Overall, these results demonstrate that a qAOP calibrated using the chemical-agnostic hierarchical approach not only provides superior model performance but also derives BML values that support robust probabilistic risk assessment.

**Figure 3:**
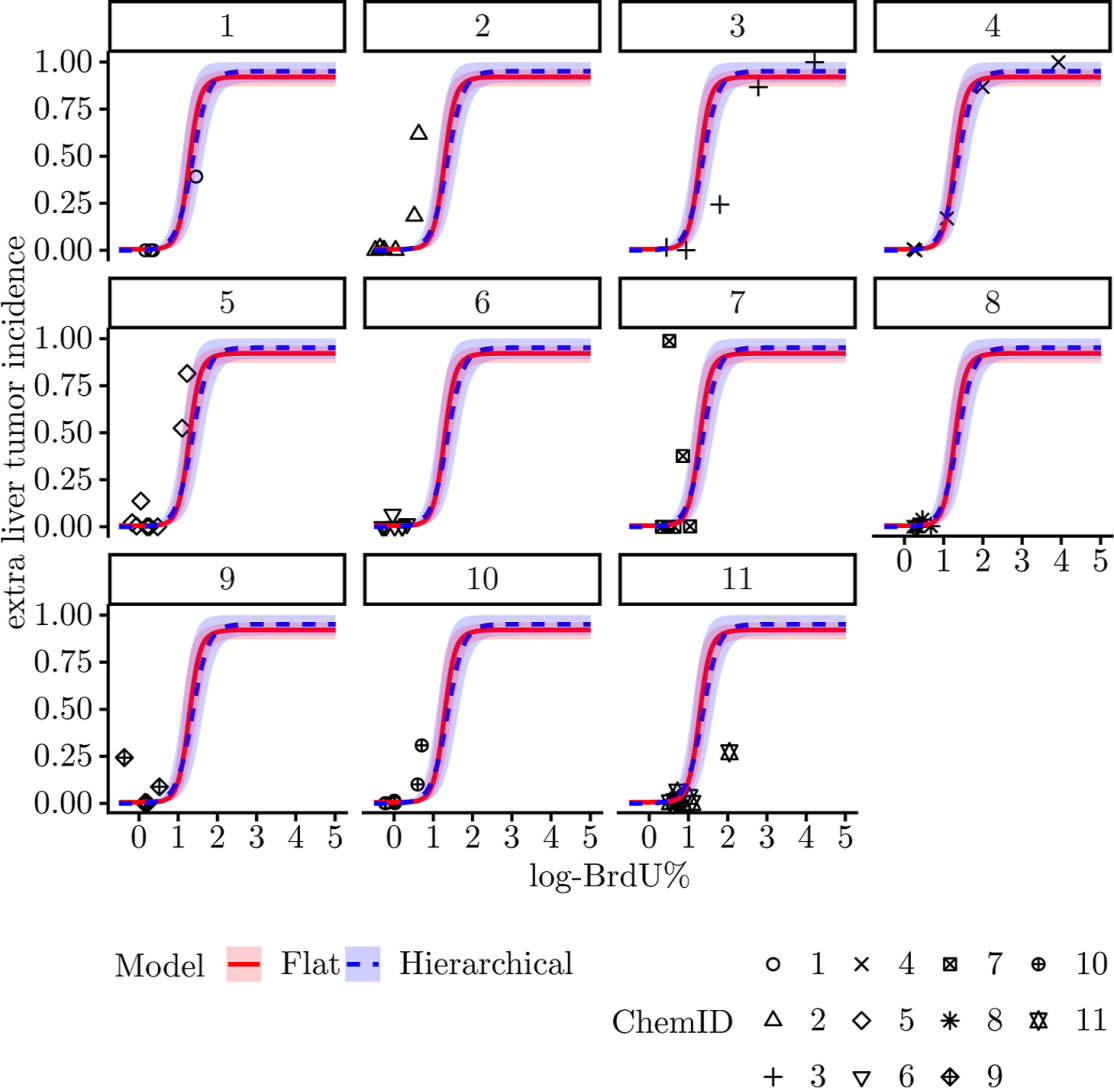
Comparing flat and hierarchical response–response models for predicting liver tumor incidence from log-BrdU%. LTI: liver tumor incidence. Ribbons are 95% posterior predictive intervals with cross-chemical variability filtered out.

## 4 Conclusions

Achieving a truly chemical-agnostic adverse outcome pathway and quantitative AOP frameworks requires explicit recognition and systematic treatment of cross-chemical heterogeneity that can confound pathway-level inferences and compromise the reliability and generalizability of qAOPs.

The chemical-agnostic calibration approach proposed in this study addresses this challenge by leveraging hierarchical structures to systematically separate chemical-specific heterogeneity from the underlying pathway effects. Through this methodological framework, chemical-specific deviations are explicitly modeled as random effects, enabling the extraction of pathway-level parameters that represent core mechanistic relationships independent of individual chemical properties.

The validity of this framework is supported by rigorous mathematical principles and demonstrated performance in a simulation study, which yields two key conclusions. First, performance differences between models with and without hierarchical calibration can be used to reveal the magnitude of heterogeneity in the data. This was shown when the hierarchical model displayed better performance if heterogeneity reached at least medium levels. Second, when heterogeneity is substantial, an uncalibrated qAOP should not be considered truly chemically agnostic in practice, as it confounds pathway-level effects with chemical-specific variation. Therefore, the hierarchical calibration approach proposed in this study represents a critical methodological advancement for developing robust and generalizable chemical-agnostic AOP frameworks to support next-generation risk assessment.

## Supplementary Material







## Notes

Glossary of acronyms, benchmark dose functions, formula and rules of interpretation for Cohen’s f effect size, prior specifications, metadata and exploratory analysis for the liver tumor case study data, fitting results on the case study including parameter posterior values and graphs, fitting results using an alternative qAOP functional form for comparison, and Stan code for the qAOP (PDF)
Veltman 2025: data for the case study; post_flat: posterior estimates of the flat model parameters; and post_hier: posterior estimates of the hierarchical model parameters (XLSX)
Scripts used in this study in computer-readable formats: test_Hill_DHill_lnorm_public.R (simulation script) and *flat and *hier.stan (flat and hierarchical models, respectively) (ZIP)

## References

[1] Villeneuve D. L., Crump D., Garcia-Reyero N., Hecker M., Hutchinson T. H., LaLone C. A., Landesmann B., Lettieri T., Munn S., Nepelska M., Ottinger M. A., Vergauwen L., Whelan M. (2014). Adverse Outcome Pathway (AOP) Development I: Strategies
and Principles. Toxicol. Sci..

[2] OECD Guidance Document for the Use of Adverse Outcome Pathways in Developing Integrated Approaches to Testing and Assessment (IATA). Organisation for Economic Co-operation and Development, 2017.

[3] Villeneuve, D. ; Meek, B. ; Viviani, B. ; Burgdorf, T. ; LaLone, C. ; O’Brien, J. ; Knapen, D. ; Angrish, M. ; FitzGerald, R. ; Tanabe, S. AOP Developers’ Handbook 2.7. https://aopwiki.org/handbooks/5 2024, accessed 9/30/2025.

[4] Spinu N., Cronin M. T. D., Enoch S. J., Madden J. C., Worth A. P. (2020). Quantitative
adverse outcome pathway (qAOP) models for toxicity prediction. Arch. Toxicol..

[5] Pallocca G., Moné M. J., Kamp H., Luijten M., Van de Water B., Leist M. (2022). Next-generation risk assessment of chemicals - Rolling out a human-centric
testing strategy to drive 3R implementation: The RISK-HUNT3R project
perspective. Alternatives to Animal Experimentation:
ALTEX.

[6] Zhou Z., Pennings J. L., Sahlin U. (2025). Causal, predictive
or observational?
Different understandings of key event relationships for adverse outcome
pathways and their implications on practice. Environ. Toxicol. Pharmacol..

[7] Meek M., Palermo C. M., Bachman A. N., North C. M., Jeffrey
Lewis R. (2014). Mode of action human relevance (species concordance) framework: Evolution
of the Bradford Hill considerations and comparative analysis of weight
of evidence. J. Appl. Toxicol..

[8] Meek M., Boobis A., Cote I., Dellarco V., Fotakis G., Munn S., Seed J., Vickers C. (2014). New developments in
the evolution and application of the WHO/IPCS framework on mode of
action/species concordance analysis. J. Appl.
Toxicol..

[9] Zgheib E., Gao W., Limonciel A., Aladjov H., Yang H., Tebby C., Gayraud G., Jennings P., Sachana M., Beltman J. B., Bois F. Y. (2019). Application
of three approaches for quantitative AOP
development to renal toxicity. Comput. Toxicol..

[10] Song Y., Zheng K., Brede D. A., Gomes T., Xie L., Kassaye Y., Salbu B., Tollefsen K. E. (2023). Multiomics
Point of Departure (moPOD) Modeling Supports an Adverse Outcome Pathway
Network for Ionizing Radiation. Environ. Sci.
Technol..

[11] Furxhi I., Murphy F., Poland C. A., Sheehan B., Mullins M., Mantecca P. (2019). Application of Bayesian
networks in determining nanoparticle-induced
cellular outcomes using transcriptomics. Nanotoxicology.

[12] Burgoon L. D., Angrish M., Garcia-Reyero N., Pollesch N., Zupanic A., Perkins E. (2020). Predicting the Probability
that a Chemical Causes Steatosis
Using Adverse Outcome Pathway Bayesian Networks (AOPBNs). Risk Anal..

[13] Gou X., Ma C., Ji H., Yan L., Wang P., Wang Z., Lin Y., Chatterjee N., Yu H., Zhang X. (2023). Prediction of zebrafish
embryonic developmental toxicity by integrating omics with adverse
outcome pathway. J. Hazard. Mater..

[14] McElreath, R. Statistical Rethinking: A Bayesian Course with Examples in R and Stan; Chapman and Hall/CRC, 2018.

[15] Gelman, A. ; Stern, H. S. ; Carlin, J. B. ; Dunson, D. B. ; Vehtari, A. ; Rubin, D. B. Bayesian Data Analysis; Chapman and Hall/CRC, 2013.

[16] Rota M., Bellocco R., Scotti L., Tramacere I., Jenab M., Corrao G., La Vecchia C., Boffetta P., Bagnardi V. (2010). Random-effects meta-regression models
for studying nonlinear dose-response relationship, with an application
to alcohol and esophageal squamous cell carcinoma. Stat. Med..

[17] Allen B., Shao K., Hobbie K., Mendez Jr W., Lee J. S., Cote I., Druwe I., Gift J., Davis J. A. (2020). Bayesian hierarchical dose-response
meta-analysis of
epidemiological studies: Modeling and target population prediction
methods. Environ. Int..

[18] U.S. EPA , Benchmark Dose Technical Guidance; EPA: 2022.

[19] More S. J., Bampidis V., Benford D., Bragard C., Halldorsson T. I., Hernández-Jerez A. F., Bennekou S. H., Koutsoumanis K., Lambré C., EFSA Scientific Committee (2022). Guidance on the use
of the benchmark dose approach in risk assessment. EFSA J..

[20] U.S. EPA , BMDS Version 3.3 User Guide (Oct 2022); EPA/600/R-21/245; EPA: Washington, DC, 2022.

[21] Weiss J. N. (1997). The Hill
equation revisited: uses and misuses. FASEB
J..

[22] Slob W., Bakker M. I., Bokkers B. G. H., Chen G., Chiu W. A., Mennes W., Nicolaie M. A., Setzer R. W., White P. A. (2025). The use
of canonical dose–response models for benchmark dose analysis
of continuous toxicological data. Crit. Rev.
Toxicol..

[23] Shao K., Zhou Z., Xun P., Cohen S. M. (2021). Bayesian Benchmark
Dose Analysis for Inorganic Arsenic in Drinking Water Associated With
Bladder and Lung Cancer Using Epidemiological Data. Toxicology.

[24] Vehtari A., Ojanen J. (2012). A survey of Bayesian
predictive methods for model assessment,
selection and comparison. Stat. Surv..

[25] Vehtari A., Gelman A., Gabry J. (2017). Practical Bayesian model evaluation
using leave-one-out cross-validation and WAIC. Stat. Comput..

[26] Vehtari, A. Cross-Validation FAQ, 2024.

[27] Vehtari, A. ; Gabry, J. ; Magnusson, M. ; Yao, Y. ; Bürkner, P.-C. ; Paananen, T. ; Gelman, A. Loo: Efficient leave-one-out cross-validation and WAIC for Bayesian Models, 2024.

[28] Stan Development Team RStan : the R Interface to Stan, 2022.

[29] Wheeler M.
W. (2023). An investigation
of non-informative priors for Bayesian dose-response modeling. Regul. Toxicol. Pharmacol..

[30] Hartmann M., Agiashvili G., Bürkner P., Klami A. (2020). Flexible prior elicitation
via the prior predictive distribution. arXiv.

[31] Cohen, J. Statistical Power Analysis for the Behavioral Sciences; routledge, 2013.

[32] Funder D.
C., Ozer D. J. (2019). Evaluating
Effect Size in Psychological Research: Sense
and Nonsense. Adv. Meth. Pract. Psychol. Sci..

[33] Veltman C. H., Khalidi H., Zgheib E., van de Water B., Luijten M., Pennings J. L. (2025). Towards a quantitative adverse outcome
pathway for liver carcinogenesis: From proliferation to prediction. Comput. Toxicol..

[34] AOP Wiki Relationship 298 Induction, Sustained Cell Proliferation Leads to Formation, Liver Tumor, 2025.

[35] AOP Wiki AOP : 32 Inhibition of iNOS, Hepatotoxicity, and Regenerative Proliferation Leading to Liver Tumors, 2023.

[36] Sivakumar S., Porter-Goff M., Patel P. K., Benoit K., Rhind N. (2004). In Vivo Labeling
of Fission Yeast DNA with Thymidine and Thymidine Analogs. Methods.

