# Supplementary material for: A Method to Calibrate Chemical-Agnostic Quantitative Adverse Outcome Pathways on Multiple Chemical Data
Source: Chem Res Toxicol. 2026 Apr 14;39(5):852–61. doi: 10.1021/acs.chemrestox.5c00435 (PMC13188161; doi:10.1021/acs.chemrestox.5c00435)
Supplement: Supplementary file 1 [file tx5c00435_si_001.pdf]

Supporting Information

A Method to Calibrate Chemical Agnostic Quantitative Adverse Outcome Pathways on Multiple Chemical Data

Zheng Zhou[1,*], Ullrika Sahlin[1]

[1] Department of Earth and Environmental Sciences, Lund University, Lund, 22362, Sweden

[*] Corresponding email: zheng.zhou@mgeo.lu.se

Contents

1 Glossary of Acronyms S2

2 Benchmark Dose Functions S2

3 Formula and Rules of Interpretation for Cohen's f Effect Size S4

4 Prior Specification S4

5 Data of Case Study and Exploratory Analysis S5

6 Case Study Fitting Results S8

7 Comparison of Flat and Hierarchical Model on Different Response-response Functional Forms S11

1 Glossary of Acronyms

- AOP: adverse outcome pathway
- AO: adverse outcome
- qAOP: quantitative adverse outcome pathway
- KE: key event
- NAM: new approach methodology
- POD: point of departure
- MOA: mode of action
- BMD: benchmark dose
- BMDS: benchmark dose software
- LOO-CV: leave-one-out cross validation
- PSIS: pareto smoothed importance sampling
- ELPD: expected log predictive density
- AIC: Akaike information criterion
- WAIC: widely applicable information criterion
- MCMC: Markov chain Monte Carlo
- BrdU: 5-bromo-2-deoxyuridine
- PDF: probability density function
- BMR: benchmark response
- BML: benchmark level

2 Benchmark Dose Functions

Candidate functions for continuous and dichotomous types of responses were adapted from the U.S. EPA Benchmark Dose Software [1].

Table S1: Continuous models and parameterizations

Model	Parameterization	Additional Specifications
Linear	f(dose)=a+b*dose;<br>a = control response;<br>b= slope	b>0 when increasing, b<0 when decreasing
Power	f(dose)=a+b*dose^g;<br>a = control response;<br>b = slope;<br>g = power	0<=g<=18;<br>g may be further restricted to g >= 1 to avoid infinite slope at the control dose.
Polynomial	f(dose)=a+b_1*dose+b_2*dose^2+...b_n*dose^n;<br>a = control response;<br>b_1,...b_n = polynomial coefficients;<br>n = degree of polynomial	The degree of polynomial should not exceed the number of dose group minus one
Hill	f(dose)=a+b*dose^g/(c^g+dose^g);<br>a = control response;<br>b = maximum change;<br>c = dose with half-maximal change;<br>g = power	It is recommended to normalize the response so that the half-maximal dose could be constrainted: 0<=c<=5<br>0<=g<=18, g may be further restricted to g>= 1
Exponential	Exp2: f(dose)=a * exp(±(b*dose));<br>Exp3: f(dose)=a * exp(±(b*dose)^d);<br>Exp4: f(dose)=a*(c-(c-1)*exp(-b*dose));<br>Exp5: f(dose) = a * (c-(c-1)*exp(-(b*dose)^d));<br>a = control response;<br>b = slope;<br>c = asymptote term;<br>d = power	a>0;<br>Direction of change is controlled by the ± sign and parameter c;<br>0<b<100;<br>0<c<1 when decrasing; c>1 when increasing;<br>1<=d<=18

Table S2: Dichotomous models and parameterizations

Model	Parameterization	Additional Notes
Quantal-Linear	p(dose)=a+(1a)×[1exp(b×dose)];<br>a = background;<br>b = slope	0<=a<=1;<br>0<b<100;
Probit	p(dose)=(a+b*dose);<br>a = intercept;<br>b = slope	b>=0
Logistic	p(dose)=1/[1+exp(-a-b*dose)];<br>a = intercept;<br>b = slope	b>=0
Multistage	p(dose)=a+(1-a)(1-exp[-\sum_{j=1}^n{b_j*dose^j}]);<br>a = background;<br>b_j = dose coefficient	j<=23;<br>0<=a<=1;<br>b_j can be stricted to b>=0 which guarantee that the model will be either flat or always increasing
Weibull	p(dose)=a+(1-a)*(1-exp[-b*dose^g]);<br>a = background;<br>b = slope;<br>g = power	0<=a<=1;<br>0<b<100;<br>0<g<=18, g can be stricted to g>=1
LogLogistic	p(dose)=a+((1-a)/{1+exp[-b-g*log(d)]});<br>a = background;<br>b = slope;<br>g = power	0<=a<=1;<br>0<g<18, g could be restricted to g>=1
LogProbit	p(dose)=a+(1-a)*[b+g*log(d)];<br>a = background;<br>b = slope;<br>g = power	0<=a<=1;<br>0<g<18, g could be restricted to g>=1
Dichotomous Hill	p(dose)=v*(1+r*exp(-(h+q*log(x))))/(1+r*exp(-(h+q*log(x))));<br>v = maximum probability;<br>v*q = background probability, q = extra risk;<br>h = potency;<br>r = power	0<v<=1;<br>0<=q<1;<br>-18<h<=18;<br>0<=p<=18, can be restricted to p>=1;

3 Formula and Rules of Interpretation for Cohen's f Effect Size

$$\begin{aligned} ES &= \sqrt{\frac{SSB}{SST - SSB}} \\ SSB &= \sum_{j=1}^{K} n_j(\mu_j - \hat{\mu})^2 \\ SST &= \sum_{i=1}^{N} (y_i - \hat{\mu})^2 \end{aligned} \tag{1}$$

where SSB and SST are between-chemical and total squared variance, respectively; $\hat{\mu}$ is global mean of the data; j is chemical index, n_j and μ_j are the size and mean per chemical. The value of Cohen's f can be interpreted as follows [2]: when f=0, there is no between-chemical differences; when f>0, f=0.1 ˜ small differences, f=0.2 ˜ medium differences, f>=0.4 ˜ large differences. The effect size values are calculated using Analysis of Covariate (ANCOVA) as the ratio of between-group and within group variances, adjusted for covariates [3]. These are rules of thumb, developed to assist the interpretation of Cohen's f effect sizes from psychological studies [3]. The values in the rules have been validated through simulation studies and updated [2, 4]. A large Cohen's f value indicates the between-group variance is bigger than the within-group variance.

4 Prior Specification

Prior choices for the simulation study. k is chemical index.

Dose-response part (shared by flat and hierarchical model):

$$\begin{aligned} \sigma_{Y_1} &\sim Cauchy(0,1) \\ a &\sim normal(0,1) \\ b &\sim normal(0,1) \\ c &\sim normal(0,5) \\ g &\sim normal(0,1) \end{aligned} \tag{2}$$

Response-response part, flat model:

$$\begin{aligned} (v,q) &\sim multivariate\_normal([0,0]',[1,1]') \\ h &\sim normal(0,1) \\ r &\sim normal(0,1) \end{aligned} \tag{3}$$

Response-response part, hierarchical model:

$$
\begin{aligned}
\mu_{vq} &\sim normal(0,1) \\
\sigma_{vq} &\sim Cauchy(0,1) \\
L_{vq} &\sim lkj\_cholesky(2) \\
z_{vq} &\sim normal(0,1) \\
\mu_h &\sim normal(0,5) \\
\sigma_h &\sim Cauchy(0,1) \\
r &\sim normal(0,1)
\end{aligned} \tag{4}
$$

5 Data of Case Study and Exploratory Analysis

From the case study of non-mutagenic tumorgenesis induced by sustained cell proliferation in liver [5]. The data is provided as Veltman2025.csv and includes the following variables:

1. N_subj: number of animals per dose group in BrdU measurements
2. Unit: reported unit of dose in the source report
3. Dose: doses from the source report, of Route at Unit
4. Unit_liver: unit of converted internal liver doses from [5]
5. Liver: converted internal liver doses from [5]
6. Mod: modifying factor to unify internal liver doses to µg/mL
7. Liver_M: unified internal liver doses (µg/mL) = Liver * Mod
8. BrdU_mean: KE_{up} BrdU percentage, group mean, corresponding to m_Y
9. BRdU_SD: KE_{up} BrdU percentage, group standard deviation, corresponding to s_Y
10. N_tumour: number of animals per dose group in liver tumor measurements
11. Extra_tumour: extra liver tumor incidence per dose group
12. ETI: extra tumor incidence
13. LogBrdUSD: BrdU_SD at log scale, corresponding to s
14. LogBrdUmean: BrdU_mean at log scale, corresponding to m

The group log-mean m and log-standard deviation s from Equation (2) can be calculated on the sample mean m_Y and sample standard deviation s_Y on the regular scale using the equation below:

$$
\begin{aligned}
m &= \sqrt{\log(1 + (s_Y/m_Y)^2)} \\
s &= \log(m_Y) - 0.5m^2
\end{aligned} \tag{5}
$$

The dose-response data [5] of the upstream and downstream KE is illustrated as Figure S1 and S2 respectively.

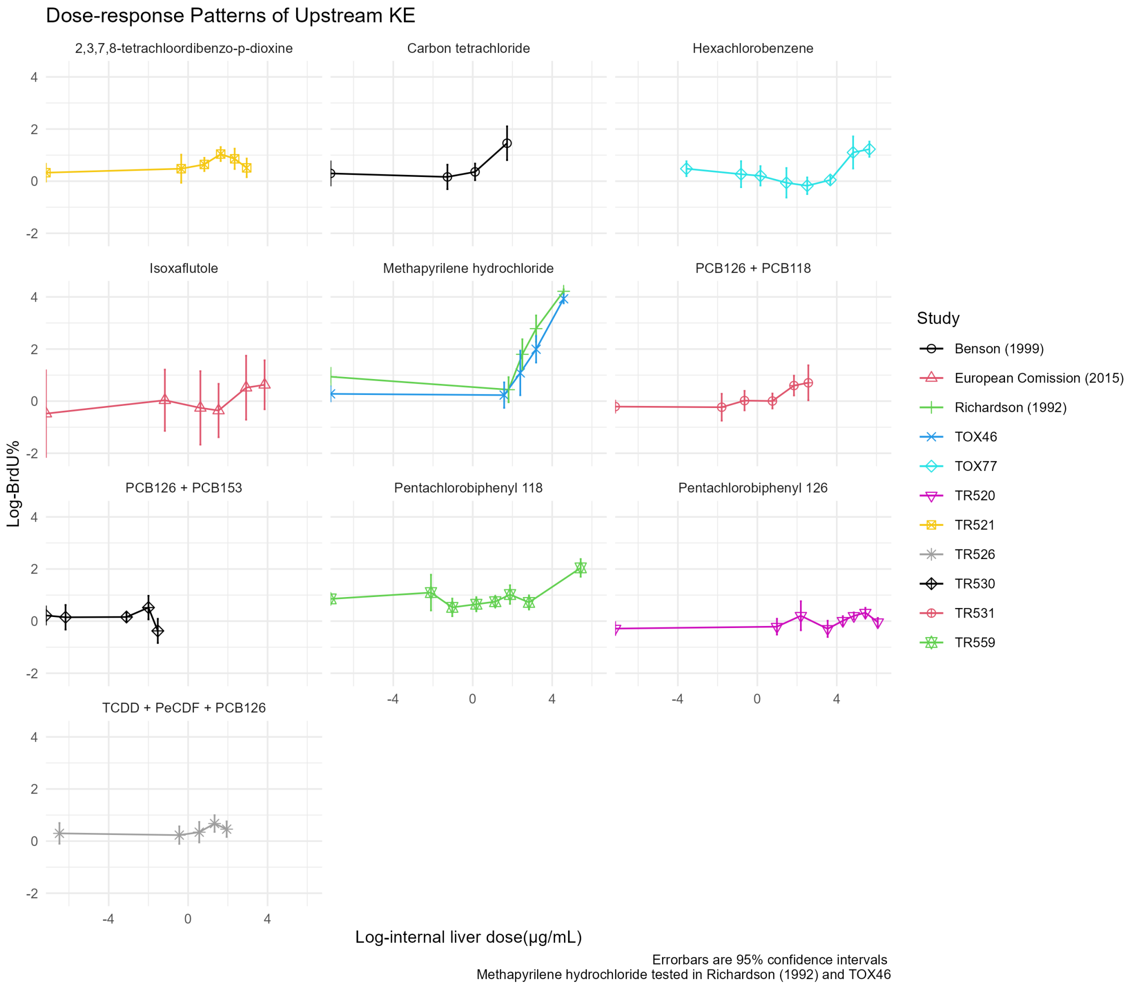

Figure S1: Upstream KE Dose-response Patterns by Chemical

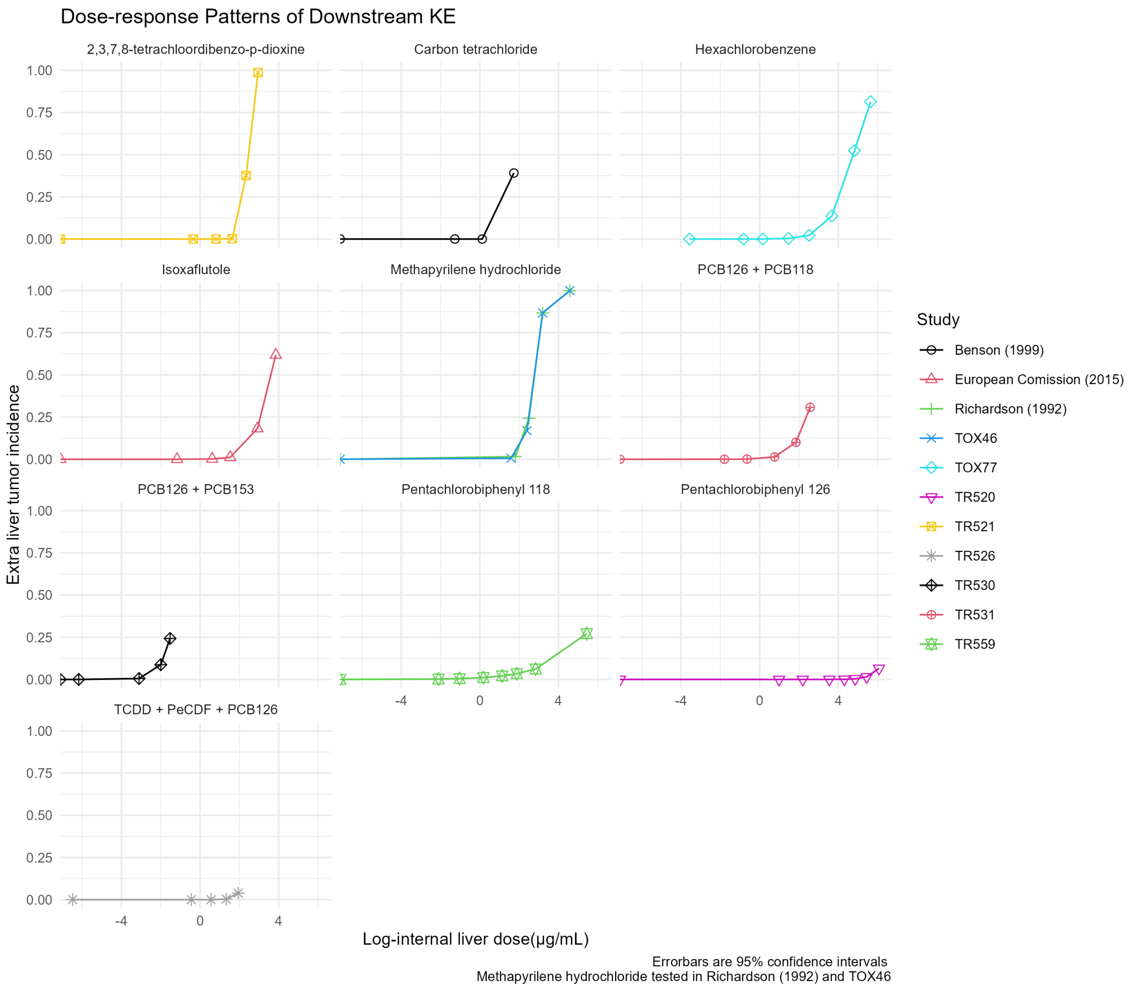

Figure S2: Downstream KE Dose-response Patterns by Chemical

The response-response data is illustrated as Figure S3.

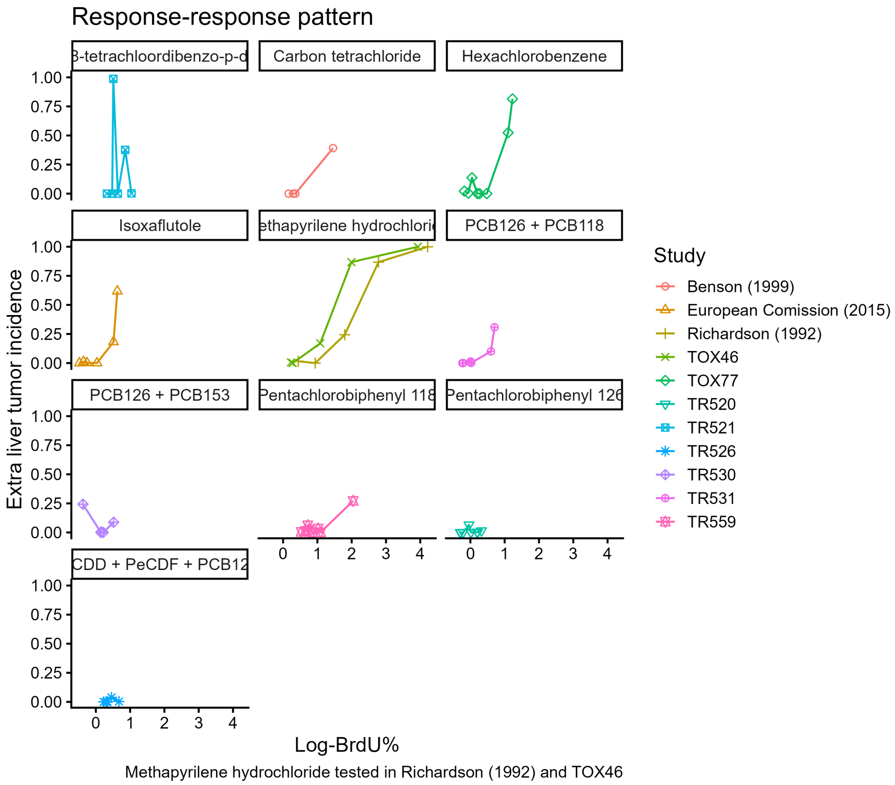

Figure S3: Response-response

Methapyrilene hydrochloride was tested from Richardson (1992) and TOX46 and visualized in the same panel. Although the overall trends and shapes are similar between them, there are significant differences which represent the variability from chemical sources and experimental conditions between the two studies. Therefore, the data from these two studies were treated as if they are different chemicals.

A constant variation (CV) test was performed to evaluate if the BrdU (KEup) data meets either the constant variation or constant coefficient of variation assumption [6, 7]. As shown in Figure S4, the per group log-standard deviation increases proportionally to the per group log-mean ($p > 0.05$). Therefore, assuming BrdU follows a lognormal distribution, the constant coefficient of variation assumption holds, which supports the modeling of σ_1 in Equation 2 in the main text as a fixed value per chemical.

6 Case Study Fitting Results

Here provides a comparison of the estimated posterior response-response parameter values between the flat and hierarchical model.

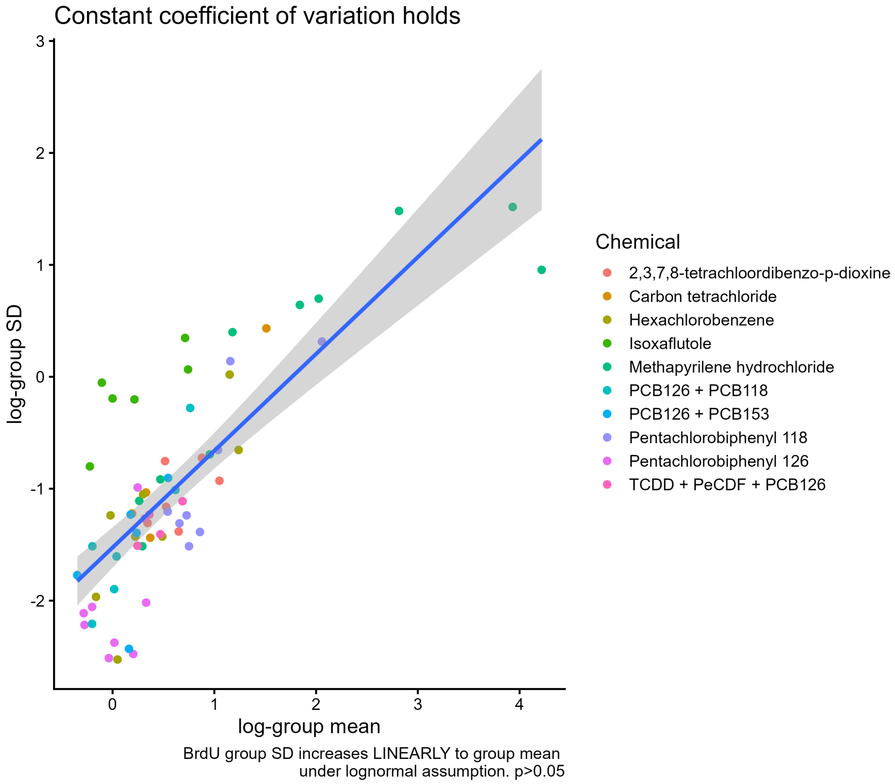

Figure S4: Constant coefficient of variation holds under lognormality for BrdU

Table S3: Comparison of Posterior Response-response Parameter Values

Model	Parameter Name	Estimate	SD	5%	95%
Flat	v	0.9193	0.0272	0.8714	0.9601
	q	0.0062	0.002	0.0033	0.0099
	h	3.6288	0.4219	3.0026	4.3755
	r	7.283	1.4214	5.2968	9.8721
Hierarchical	v	0.9471	0.0963	0.8261	0.9993
	q	0.0018	0.0612	0	0.0678
	h	3.9603	0.5469	3.0934	4.9524
	r	5.6766	0.8892	4.336	7.4213

The figure below visualizes the chemical-specific random effects from the hierarchical response-response model for Y_2, which was overlooked by the flat model. A model in each panel is a conditional prediction specific to the chemical. The magnitude of these random effects reflects the heterogeneity level, which was overlooked by the flat model. These random noises should be excluded when applying the hierarchical model for deriving points of departures for new chemicals.

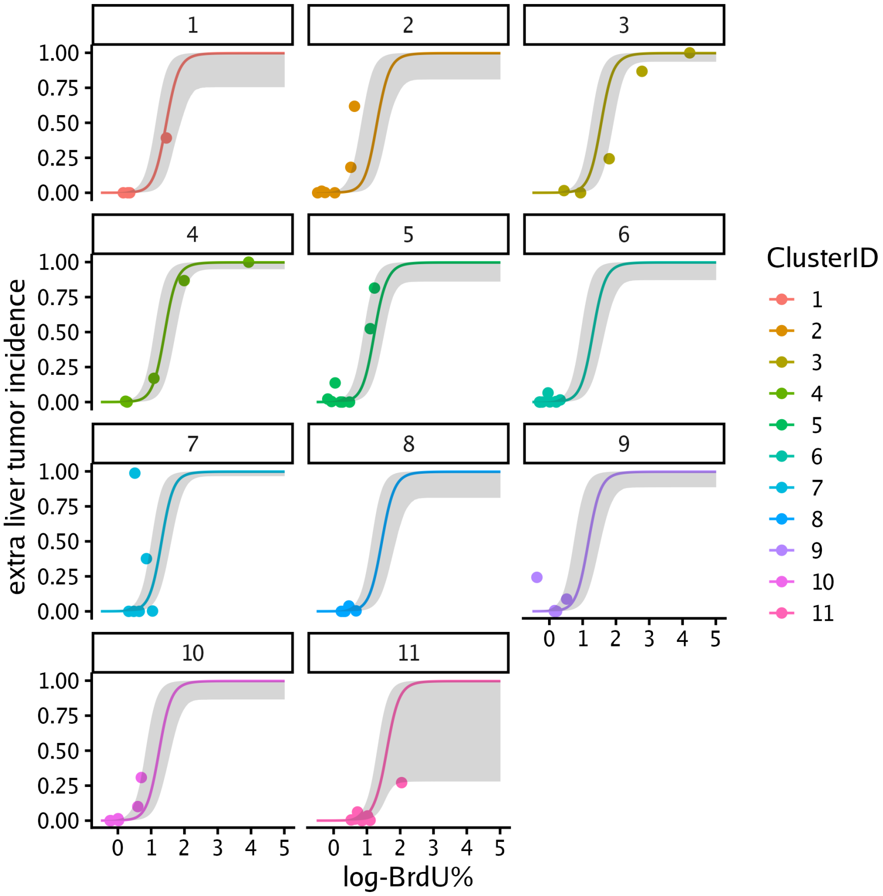

Figure S5: Chemical-specific Random Effects in Response-response of Liver Tumor Incidence Predicted by Log-BrdU% by the Hierarchical Model

7 Comparison of Flat and Hierarchical Model on Different Response-response Functional Forms

The case study comparison between the flat and hierarchical model was also performed using different functional forms and parameterizations on the response-response for Y_2. In summary, the results were comparable to that in the main text that given the low-to-medium data heterogeneity, the hierarchical model performs better than the flat model. These results support the key conclusion of our calibration approach that the improvement of calibration with hierarchical models depends on the data heterogeneity and not sensitivie to the choice of functional forms and parameterizations.

The dichotomous Hill equation from EPA BMDS [1] with parameters $\theta_2 = \{v, q, h, r\}$ was used (Equation (6)):

$$f_2(Y_1, \theta_2) = v \frac{1 + q e^{-(h + r \log(Y_1))}}{1 + e^{-(h + r \log(Y_1))}} \tag{6}$$

where $0 < v \leq 1$ is maximum probability; $0 \leq q < 1$ is background risk so that vq is background probability; h is potency, or half-maximal effect level; $r \geq 0$ is power, i.e., steepness of sigmoid curves.

The fitted response-response curves (Figure S6) supports the results of model comparison that the hierarchical model has marginally better predictive performance than the flat model (WAIC_diff = 13.2(6.1) and LOO_diff = 18.2(6.7)).

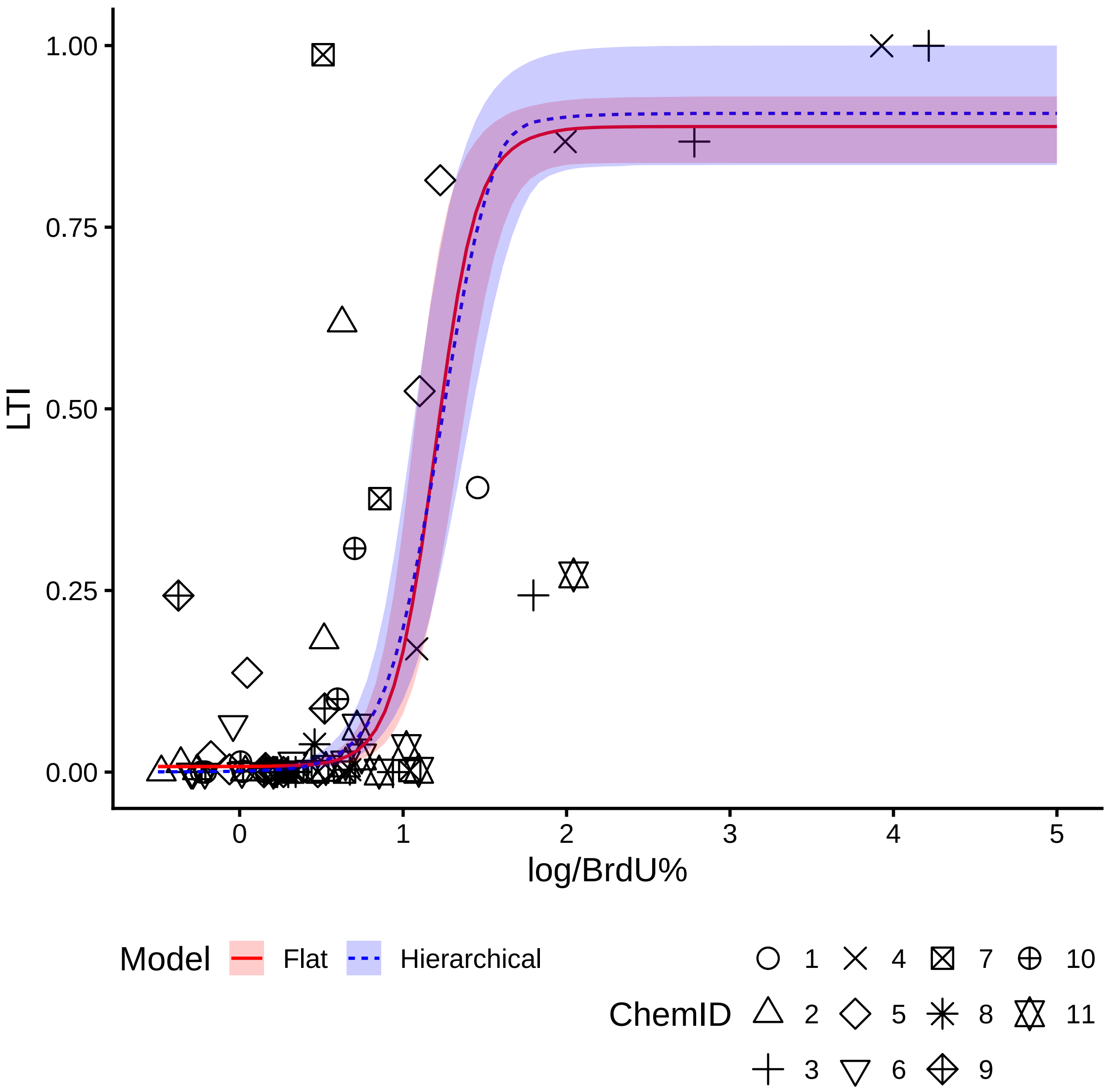

Figure S6: Comparing Flat and Hierarchical Response-response Models for Predicting Liver Tumor Incidence from Log-BrdU%. LTI: liver tumor incidence. posterior predictive intervals with cross-chemical variability filtered out

The results by this qAOP with alternative functional forms are completely consistent with the main text. It supports that the impact of calibration depends only on the level of heterogeniety in the data but not the functional forms.

References

(1) U.S. EPA, *Benchmark Dose Technical Guidance*; EPA: 2022.

(2) Funder, D. C.; Ozer, D. J. Evaluating Effect Size in Psychological Research: Sense and Nonsense. *Advances in Methods and Practices in Psychological Science* **2019**, *2*, 156–168.

(3) Cohen, J., *Statistical power analysis for the behavioral sciences*; routledge: 2013.

(4) Sawilowsky, S. S. New Effect Size Rules of Thumb. *Journal of Modern Applied Statistical Methods* **2009**, *8*, DOI: `10.22237/jmasm/1257035100`.

(5) Veltman, C. H.; Khalidi, H.; Zgheib, E.; van de Water, B.; Luijten, M.; Pennings, J. L. Towards a quantitative adverse outcome pathway for liver carcinogenesis: From proliferation to prediction. *Computational Toxicology* **2025**, 100359.

(6) U.S. EPA, *BMDS Version 3.3 User Guide (Oct 2022)*; EPA/600/R-21/245; EPA: Washington, DC, 2022.

(7) EFSA Scientific Committee et al. Guidance on the use of the benchmark dose approach in risk assessment. *EFSA Journal* **2022**, *20*, e07584.
